# An Overview of the Role of MicroRNAs on Carcinogenesis: A Focus on Cell Cycle, Angiogenesis and Metastasis

**DOI:** 10.3390/ijms24087268

**Published:** 2023-04-14

**Authors:** Leonel Pekarek, Diego Torres-Carranza, Oscar Fraile-Martinez, Cielo García-Montero, Tatiana Pekarek, Miguel A. Saez, Francisco Rueda-Correa, Carolina Pimentel-Martinez, Luis G. Guijarro, Raul Diaz-Pedrero, Melchor Alvarez-Mon, Miguel A. Ortega

**Affiliations:** 1 Department of Medicine and Medical Specialities, Faculty of Medicine and Health Sciences, University of Alcalá, 28801 Alcala de Henares, Spain; 2 Ramón y Cajal Institute of Sanitary Research (IRYCIS), 28034 Madrid, Spain; 3 Oncology Service, Guadalajara University Hospital, 19002 Guadalajara, Spain; 4 Pathological Anatomy Service, Central University Hospital of Defence-UAH Madrid, 28801 Alcala de Henares, Spain; 5 Unit of Biochemistry and Molecular Biology, Department of System Biology (CIBEREHD), University of Alcalá, 28801 Alcala de Henares, Spain; 6 Department of Surgery, Medical and Social Sciences, Faculty of Medicine and Health Sciences, University of Alcalá, 28801 Alcala de Henares, Spain; 7 Department of General and Digestive Surgery, General and Digestive Surgery, Príncipe de Asturias Teaching Hospital, 28805 Alcala de Henares, Spain; 8 Immune System Diseases-Rheumatology, Oncology Service an Internal Medicine (CIBEREHD), University Hospital Príncipe de Asturias, 28806 Alcala de Henares, Spain; 9 Cancer Registry and Pathology Department, Principe de Asturias University Hospital, 28806 Alcala de Henares, Spain

**Keywords:** microRNA (miRNAs), angiogenesis, cell cycle, lymphangiogenesis, metastasis

## Abstract

In recent years, the importance of epigenetic markers in the carcinogenesis of different malignant neoplasms has been demonstrated, also demonstrating their utility for understanding metastatic spread and tumor progression in cancer patients. Among the different biomarkers, microRNAs represent a set of non-coding RNAs that regulate gene expression, having been involved in a wide variety of neoplasia acting in different oncogenic pathways. Both the overexpression and downregulation of microRNAs represent a complex interaction with various genes whose ultimate consequence is increased cell proliferation, tumor invasion and interaction with various driver markers. It should be noted that in current clinical practice, even though the combination of different microRNAs has been shown to be useful by different authors at diagnostic and prognostic levels, there are no diagnostic kits that can be used for the initial approach or to assess recurrences of oncological diseases. Previous works have cited microRNAs as having a critical role in several carcinogenic mechanisms, ranging from cell cycle alterations to angiogenesis and mechanisms of distant metastatic dissemination. Indeed, the overexpression or downregulation of specific microRNAs seem to be tightly involved in the modulation of various components related to these processes. For instance, cyclins and cyclin-dependent kinases, transcription factors, signaling molecules and angiogenic/antiangiogenic products, among others, have been recognized as specific targets of microRNAs in different types of cancer. Therefore, the purpose of this article is to describe the main implications of different microRNAs in cell cycle alterations, metastasis and angiogenesis, trying to summarize their involvement in carcinogenesis.

## 1. Introduction

Cancer is a global health concern with a huge impact on our society. Epidemiological data provided by GLOBOCAN show that the incidence of cancer in the world was approximately 19.3 million new cases by the year 2020 and there were 10 million deaths in the same period [1]. The malignization process from a normal functioning cell to a tumor invasive neoplastic cell is the consequence of a set of alterations in the control of the cell cycle, invasion capacity and blood and nutrient supply that generate uncontrolled cell growth, the activation of tissue invasion mechanisms and alterations in peritumoral blood and lymphatic vessels [2]. Currently, it is known that carcinogenesis events are mainly driven by epigenetic and genetic variations which lead to changes in transcriptional and biological processes in tumoral cells [3]. MicroRNAs (miRNAs) are small non-coding RNA molecules, about 20 nucleotides in size, that constitute a large number of post-transcriptional regulatory genes. Their implications in different pathways of cell differentiation, proliferation and apoptosis have been described by numerous authors acting as endogenous epigenetic factors promoting or limiting the expression of a gene after transcription [4]. It should be noted that a single miRNA can regulate the transcription of hundreds of different genes [5], forming a complex network, aiding the understanding of plenty of cellular mechanisms under healthy and diseased conditions [6,7].

### 1.1. A Short View on microRNAs Biology

Regarding their biogenesis process, miRNAs are generally produced in the nucleus following two major mechanisms: canonical and noncanonical pathways. The former is mediated by the transcription of a primary miRNA (pri-miRNA) due to the action of RNA polymerases II or III [8,9]. Then, this pri-miRNA is further processed into a precursor miRNA (pre-miRNA) by a ribonuclease named Drosha and a double-stranded RNA-binding protein, known as DiGeorge Syndrome Critical Region 8 (DGCR8), eventually being exported by the exportin 5 (XPO5)/RanGTP complex from the nucleus to the cytosol where an RNase III endonuclease (Dicer) transforms pre-miRNA into a 22-nucleotide miRNA duplex [10,11]. Afterwards, miRNA maturation concludes with the formation of the ribonucleoprotein complex known as miRISC (miRNA-induced silencing complex), which is composed of one strand of the miRNA (guide strand), Dicer, PACT (protein activator of Protein Kinase R), TRBP (transactivation response element RNA-binding protein) and Argonaute (Ago) proteins [12]. The complex binds to the mRNA, activating the above-mentioned mechanisms and repressing gene expression. The other miRNA strand (passenger strand) is often (but not always) removed. Non-canonical pathways include Drosha/DGCR8 and Dicer-independent manners. In this sense, some introns (named mirtrons), endogenous short-hairpin RNAs (shRNAs), small nucleolar RNAs (snoRNAs) and transfer RNAs (tRNAs) are some elements involved in the non-canonical biogenesis of miRNAs, although further mechanisms have also been described [13]. In addition, there are some miRNAs that are synthesized in the mitochondria of cells, named as mitochondrial miRNAs (mito-miRs), whose relevance in cancer is being increasingly demonstrated [14]. The main targets of miRNAs are located in the cytoplasm. However, there is also evidence of miRNA/mRNAs silencing in the rough endoplasmic reticulum (RER), the trans Golgi network (TGN), early and late endosomes, lysosomes, stress granules, processing bodies and multivesicular bodies, and also in the proper nucleus and mitochondria [10]. In turn, it is important to understand that miRNA biogenesis is under precise spatial and temporal regulation, and it may be regulated at various levels [15]. Hence, miRNAs represent critical epigenetic modulators of gene expression by influencing a set of products, and due to their relevance the biogenesis and function of these agents are strongly regulated by cells.

### 1.2. microRNAs in Cancer

Hanahan and Weinberg [16] described the hallmarks of cancer considering the main biological features of cancer: (1) the sustaining of proliferative signaling; (2) the evasion of growth suppressors; (3) cell death avoidance; (4) the enabling of replicative immortality; (5) the inducing of angiogenesis; (6) the activation of invasion and metastasis; (7) the deregulation of cellular energetics; (8) the evasion of the immune system; (9) tumor-promoting inflammation and (10) genome instability and mutation. Because of the central role of miRNAs in the post-transcriptional regulation of protein expression and the fact that a single miRNA can target a wide variety of products, a growing body of evidence supports the relevance of miRNAs in the understanding of the carcinogenic process [17]. Indeed, it is broadly accepted that miRNAs can act as oncogenes (named oncomiRs) or tumor suppressors (tsmiRs) under certain conditions, having been shown to affect the totality of the hallmarks of cancer [18,19]. In this sense, significant alterations in miRNA biogenesis and functions have been described in tumoral cells, due to alterations in the expression and action of transcription (P53, PPARy and SAMD4) and epigenetic factors (hypermethylation and acetylation) which appear to induce miRNA overexpression or downregulation [20]. Other different mechanisms involved in miRNA dysregulation also include amplifications or deletions in the loci of different oncogenes [21,22]. Then, miRNAs act coordinately with all of the different factors, driving toward tumor growth, invasion and chemotherapy resistance, or favoring the epithelial-mesenchymal transition among other mechanisms [23,24,25]. Additionally, the effect of miRNAs in tumor cells can be different depending on the tissue, since the same miRNA can act as a tumor suppressor in one tissue and as an oncogenic signal in another [18]. Because of this, miRNAs are being placed at the forefront of translational research, representing promising therapeutic targets or agents [26], as well as diagnostic, prognostic or predictive biomarkers [27]. For example, the therapeutic targeting of miRNAs can impact the natural history of melanomas by enhancing sensitivity to both standard therapies and immune checkpoint inhibitors. In particular, elevated levels of miRNA-221 have been identified in early melano-mas, compared to healthy individuals. Increased levels have also been linked to an increased stage of disease. Moreover, circulating miRNA-615–3p levels are consistently efficient in detecting melanoma patients who have developed a progressive disease whilst being treated with immune checkpoint inhibitors [27,28]. In the following sections, we will only focus on the role of miRNAs as critical modulators of the cell cycle, angiogenesis and metastasis, although their relevance and possibilities in cancer are notably more numerous than a single manuscript can cover.

## 2. Role of microRNAs in Cell Cycle

Most cellular events, including DNA duplication, gene transcription, protein translation and the post-translational modification of proteins, occur in a cell-cycle-dependent manner [29]. Cell cycle disturbances are responsible for exacerbations in cell proliferation which characterizes cancer and a loss in cell cycle checkpoint control which promotes genetic instability, with both being considered to be major hallmarks of cancer [30,31]. In a simple manner, the cell cycle is the time that elapses since the cell divides until it re-enters in another mitotic division. The cell cycle is divided into different phases, named G1 (gap phase 1), S (DNA synthesis), G2 (gap phase 2) and M (mitosis/meiosis), eventually resulting in the formation of two daughter cells. In G1, the cell increases in size and initiates the transcription of cell cycle control genes, leading to the synthesis of various proteins and a global check before DNA duplication, a process which occurs in the S phase. At the G2, after the replication of the entire genome, the cell prepares for division, checking that no errors have been produced after the S phase [32]. Additionally, cells can enter temporally or permanently in a G0 (gap phase 0), in which cells are neither growing nor proliferating. Cell cycle progression is controlled by cyclin-dependent kinases (CDKs) and their regulatory cyclin subunits. For instance, cyclin D forms a complex with CDK4 and CDK6 for entry in G1, whereas cyclin E forms a complex with CDK2 to regulate progression from G1 into S phase. Cyclin A-CDK2 is required for fulfilling the S phase, although for the late G2 and early M phase, Cyclin A complexes with CDK1. Eventually, mitosis is further regulated by the cyclin B-CDK1 complex [33]. In a broader context, growth-factor-initiated signaling pathways (such as mitogen-activated protein kinase (MAPK) or phosphatidylinositol 3 kinase (PI3K)/Akt/mTOR pathways) and a set of transcription factors such as E2F, c-myc or p53, as well as other critical regulators such as proteins from the retinoblastoma (Rb) family, are responsible for modulating the activity of various CDK-cyclin complexes, hence defining the progression of the cell cycle [34,35,36].

miRNAs are pivotal regulators of the cell cycle and previous work has evidenced how aberrations in miRNA expression may contribute to tumor development by perturbing critical cell cycle regulators [37]. For instance, miRNAs can act via the direct targeting of cyclins and CDKs, as well as by acting coordinately with the aforementioned transcriptional factors, their modulators or growth-factor-initiated signaling pathways [38]. In this regard, a great variety of miRNAs have been identified as major regulators of the cell cycle, including the miR-15a/16, miR-17/92, miR-106b/25 and miR-221/222 clusters together with the let-7 and miR-34 families [38,39].

Most works have focused on describing miRNA dysregulation and its association with an altered cell cycle according to the type of cancer. Loh et al. [40] claimed the relevance of miR-497, miR-16, miR-30c-2-3p and miR-483-3p downregulation in breast cancer, as these miRNAs were directly implicated in the targeting of cyclin E1. Apart from this, they recognized a central role of three oncomiRs (miR-1207-5p, miR-492 and miR-135b) and a total of fifteen tumor-suppressor miRNAs (including the proper miR-497, miR-16, miR-30c-2-3p and miR-483-3p previously mentioned) to be clearly implicated in the modulation of various products involved in cell cycle dysregulation and sustained cellular proliferation. In a recent review conducted by Fariha et al. [41], 56 miRNAs were recognized as being major regulators of the cell cycle in lung cancer. Of them, 17 were identified as oncomiRNAs, whereas the remaining 39 were tumor suppressors. Additionally, these miRNAs were differentially dysregulated depending on the subtype of lung cancer and modulated the expression of many of the aforementioned targets, opening promising therapeutic opportunities. Mullany et al. [42] described in colorectal cancer patients a set of miRNAs involved in the regulation of mRNA translation at multiple levels within the cell cycle. More specifically, the transcription of the miRNAs in the paralogous clusters miR-17~92, miR-106a~363 and miR-106b~25 appeared to regulate the G1-S phases and the G1-to-S transition, through E2F and c-myc feedback and feed-forward loops. In the event of pancreatic adenocarcinomas, up to 158 miRNAs have been found to be importantly altered in these types of tumors, emphasizing the role of some of them in cell cycle dysregulation [23]. In particular, previous reviews have described the modulatory action of multiple miRNAs in several targets involved in the cell cycle. In this sense, various oncomiRs have been demonstrated to be overexpressed, including miR-21 (an inhibitor of PTEN—Phosphatase and tensin homolog), miR-155 (targeting of p53) and miR-424-5p (downregulates SOCS6 and stimulates the Ras-ERK pathway to favor tumor proliferation), whereas the decreased expression of tsmiRs such as miR-203 (involved in the loss of regulation of cell proliferation in the G1 phase due to alterations in SOCS3) is also implicated in cell cycle dysregulation [43]. On the other hand, miR 221 and miR 222 act on different kinase-dependent cyclin inhibitors such as p27 kip1 in nasopharyngeal carcinomas, which causes the hyperactivation of cyclins with increased tumor growth [44]. Similarly, other works have demonstrated that miR-16, a product downregulated in prostate cancer cells, can directly target AKT3, thus ameliorating cell cycle dysregulation and inducing cell apoptosis [45]. According to recent reviews on penile cancer [46], an overexpression of oncogenic miRNAs (miR-223-3p, miR-107 and miR-21-5p) was directly involved in PTEN suppression in addition to alterations in the mitogen-activated protein kinase pathway MAPK (ERK1/ERK2), whereas miR-145 downregulation drove to marked alterations in oncogenic genes such as c-myc. 

**Figure 1:**
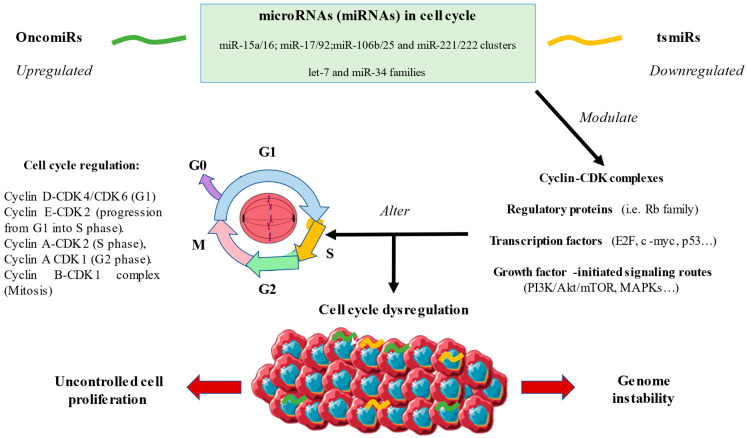
A general overview of the role of microRNAs in the cell cycle. As shown, various dysregulated oncogenic miRNAs (OncomiRs) and tumor-suppressive miRNAs (tsmiRs) are able to modulate a set of products involved in the control and progression of the cell cycle, therefore leading to cell cycle dysregulation. Consequently, two major hallmarks of cancer are promoted: uncontrolled cell proliferation and genome instability.

Collectively, cell cycle alterations are directly involved in sustained cell proliferation and genomic instability, two recognized hallmarks of cancer. miRNAs seem to play a major role in cell cycle dysregulation by acting at multiple points, as is shown in Figure 1. In Table 1, we summarize the main miRNAs altered in the cell cycle and the type of cancer in which they appear. Because of this, an increasing number of works are studying their potential applications as translational biomarkers for use in prognosis or as predictive responses to the therapy received, as well as possible therapeutic targets to improve the clinical management of these patients [38,47].

## 3. microRNAs on the Angiogenesis Process

The angiogenesis process is based on the creation of new blood vessels with the remodeling of the tissue matrix, whereas lymphangiogenesis consists of the creation of new lymphatic vessels, with these processes aimed at increasing the supply of oxygen and nutrients to the tumor cells as well as facilitating the removal of waste products [48]. Ultimately, angiogenesis and lymphangiogenesis are equally required to promote the further proliferation and dissemination of tumoral cells [49]. Numerous products have been implicated in the modulation of angiogenesis and lymphangiogenesis, with the family of the vascular endothelial growth factor (VEGF) being the most relevant modulator of both processes [48,49]. Compelling evidence in recent years supports how there is a relationship between miRNAs with different routes that condition the aberrant growth of neovessels, being able to regulate the essential signaling pathways involved in these processes such as the proper VEGF family or Notch [50]. 

This has significant implications for metastatic disease, as angiogenesis is a key factor in tumor growth and progression. Several miRNAs have been identified that can either positively or negatively regulate angiogenesis, and they are designed as AngiomiR [51]. For example, studies have shown that miR-21 upregulation promotes angiogenesis and enhances metastatic capacity by controlling several angiogenesis-related pathways, such as vascular endothelial growth factor (VEGF), transforming growth factor beta (TGF-β) and hypoxia-inducible factor (HIF) signaling. In particular, miR-21 has been shown to upregulate VEGF, TGF-β and HIF-1α at the post-transcriptional level, resulting in increased angiogenesis, which is associated with tumor growth and metastasis [52]. Additionally, miR-21 also modulates the expression of other angiogenic regulators, such as matrix metalloproteinases (MMPs) and angiopoietin-1 (Ang-1) [53,54]. On the other hand, miR-126, which is downregulated in different tumors, has been shown to lead to a decrease in the production of vascular endothelial growth factor (VEGF), whereas this miRNA also seems to be involved in the targeting of several anti-angiogenic factors, such as thrombospondin-1 (TSP1) [55,56]. Likewise, prior works have observed how the overexpression of miR-199a-3p is related both in vivo and in vitro in hepatocarcinoma cell lines, and inhibits the secretion of VEGF, VEGFR, HGF and MMPs in endothelial cells [57]. Wurdinger et al. [58] have also observed how an overexpression of miR 296 is related to the substrate related to the tyrosine kinase regulated by hepatocyte growth factor (HGS) in patients with brain gliomas, which decreases the activation cascade of release of angiogenic factors such as VEGFR2 and PDGFRβ. In addition, miR-296 has been shown to block the expressions of several pro-angiogenic signals involved in tumor growth, such as VEGF, PDGF and TNF [59]. Similarly, Fan et al. have also observed how inducing an overexpression of miR-29-c can promote the downregulation of VEGF in gliomas, which generates a lower rate of angiogenesis and invasion in this neoplasia [60]. 

We must emphasize that other critical regulators of angiogenesis in different tumors are HIF-1α and HIF-2α, upstream modulators of VEGF, activated in hypoxic situations or concomitantly by different oncogenes such as the p53, PTEN or VHL (von Hippel–Lindau) gene [61]. In this sense, authors such as Cha et al. [62] have demonstrated in lung adenocarcinoma cell lines how a downregulation of miR-519c led to an increase in the expression of HIF-1a and with it an increase in the angiogenic activity of tumor cells. The relationship of miRNAs with HIF has been evidenced by other authors such as Zhang et al. [63] in patients with neuroblastoma, observing how a downregulation of miR-145 is related to an overexpression of HIF 2-α. In prostate cancer, various proangiogenic and antiangiogenic factors modulated by miRNAs have been described [64]. For example, an overexpression of miR-30d and miR-323 increases VEGF synthesis and secretion by prostate cancer cells, and thus enhances VEGF-mediated angiogenesis in prostate cancer. miR-296 controls the levels of VEGF and PDGF receptors in angiogenic endothelial cells, while miR-182 regulates the activation of HIF-1α- and HIF-1α-moderated angiogenesis. The reduced activation of miR-146a was described in castration-resistant prostate cancer, where it controls the expression of EGFR and MMP2 in prostate cancer tissue [64]. 

Other studies have shown that miR-145 inhibited tumor angiogenesis by targeting several pro-angiogenic genes, including VEGFA, EGFR and MMP2. Furthermore, miR-145 was found to regulate the expression of genes involved in inflammation, such as TNFα, IL-1 and IL-6 [65,66,67]. These results suggest that miR-145 may play an important role in regulating tumor angiogenesis and metastatic disease. On the other hand, other works have evidenced how a downregulation of miR 17-92 is accompanied by an increase in the expression of p53 repressor transcription factors under hypoxic situations, favoring the evasion from cell death via hypoxia [68]. Another of the limiting factors in angiogenesis is based on the existence of remodeling mechanisms in angiogenesis processes such as metalloproteins, where different authors have shown how a downregulation of miR-9 and an overexpression of mir 181-5p are related to the levels of expressed MMP-14, and therefore to increased angiogenesis in breast tumors or neuroblastomas [69,70]. Other lines of study have also validated the pro/antiangiogenic role of miRNAs from the Let-7 family, miR-10b, mir-15a/16, miR-24, miR-132, miR-192, miR-195, miR-210, miR-221, miR-320 and miR-378, targeting a set of molecules involved in endothelial cell interactions, tumor cell motility and the invasion of the surrounding tissue via vessel co-option, vessel sprouting, vasculogenic mimicry and promoting the differentiation of fibroblasts into cancer-associated fibroblasts (CAFs), among other processes [51]. Therefore, miRNAs are involved in various mechanisms of angiogenesis and represent a fundamental process in the tissue invasion mechanism of different neoplasms (Table 2).

## 4. microRNAs and Metastasis

Metastatic spread is a form of tumor progression and is the final stage of any neoplastic lesion. It implies the invasion of different organs with their malfunctioning, a tendency to hemorrhage, tumor compression or different mechanisms whose growth causes the death of patients. Currently, the understanding of the mechanisms of metastasis and tumor dissemination represents the greatest frontier of progression in oncology [71,72,73]. The proposed hallmarks of metastasis are (1) motility and invasion, including here a critical mechanism known as epithelial mesenchymal transition (EMT); (2) microenvironment modulation of the local or secondary sites; (3) plasticity and (4) colonization of secondary tissues [74]. 

The pivotal role of miRNAs in the metastasis process (metastamiRs) is increasingly being demonstrated with scientific evidence, although their role in metastasis is often complex and hard to decipher [75]. For example, MiR-21 has been shown to enhance the ability of cancer cells to resist apoptosis and promote angiogenesis and tumor growth in metastatic disease. Studies have demonstrated that miR-21 expression is significantly upregulated in many cancer types, including prostate cancer and non-small cell lung cancer [76]. In breast cancer patients, increased miR-21 expression was identified as an independent prognostic biomarker for worse overall survival [77]. In this line, recent studies have demonstrated that miR-210 is overexpressed in several cancer types, including breast, prostate, colon and gastric cancer [78]. In addition, it has been found to be upregulated in the cells of metastatic disease and to act as an oncogene, promoting cell proliferation, angiogenesis and invasion. Furthermore, it may also promote the formation of premetastatic niches and metastasis in distant organs [79]. Several studies have evaluated the effect of MiR-210 on metastasis in various cancer types. For example, a meta-analysis by Hong et al. demonstrated that the overexpression of MiR-210 is related with poor survival in patients with breast cancer [80]. On the other hand, miR-199a-3p has been found to be overexpressed in multiple types of metastatic cancers including hepatocellular carcinoma, colorectal cancer, non-small cell lung cancer and breast cancer, and to play an important role in tumor progression, metastasis and chemoresistance [81]. In this line, miR-199a-3p has been found to be involved in regulating several pathways that are associated with tumor progression, such as the PI3K/Akt pathway, MAPK pathway and Wnt signaling pathway, and it has been reported that an altered expression of mir-199a-3p is associated with a poor prognosis in certain types of cancer, such as breast cancer and hepatocellular carcinoma [82,83,84]. Other miRNAs, such as miR-9 expression, are increased in gliomas, and their expression is associated with more aggressive tumor behavior [85]. Furthermore, miR-9 has been shown to be involved in the regulation of several genes associated with angiogenesis and metastasis, such as E-cadherin or VEGF, and to modulate the activity of several pathways involved in metastasis, including the PI3K/Akt and MAPK pathways [86,87]. Other studies have shown that miR-375 expression is downregulated in several tumors, including prostate cancer, glioblastoma or hepatocellular carcinoma. In addition, it was observed that miR-375 can target and reduce the expression of genes that are involved in the cascade of EMT [88,89,90]. Another example is miR-10b, a critical mediator of metastatic diseases, whose deregulation has been associated with several carcinogenic diseases, including breast cancer and hepatocellular carcinoma. Other authors have shown how an overexpression of miR 10b is accompanied by an ultimate expression of oncogenic products such as PTEN or DYRK1A (dual-specificity tyrosine phosphorylation-regulated kinase) in addition to RAS in lung cancer cell lines [91,92]. Thus, miR 10b has been shown to promote cell migration and invasion through various target genes and pathways, such as the proper PTEN/Akt, NF-κB signaling and EMT, and it has been demonstrated to be involved in tumor progression, angiogenesis and metastasis [93,94,95]. There are other studies which have demonstrated how miR-200 family members appear to exert a dual role in the invasion–metastasis cascade by suppressing EMT, tumor invasion and dissemination at the primary sites, but promote colonization in distant anatomic sites [96]. The relationship between the miR-200 family and metastasis is a topic of considerable research interest. The miR-200 family consists of five highly conserved members, including miR-200a, miR-200b, miR-200c, miR-141 and miR-429. Studies have shown that these miRNAs have proven to have efficacy in impairing tumor invasion and metastasis, through downregulating multiple transcription factors responsible for EMT. In addition, they have been proposed to function as a cluster, forming a single functional unit that modulates the invasion and metastasis of cancer cells by simultaneously targeting multiple pathways, such as WNT/beta-catenin signaling and TGF-beta/Smad3 signaling [97,98,99,100,101]. Other authors have shown that postoperative plasma miR-141 is a suggested biomarker of colorectal cancer recurrence after surgical resection. It has also been reported that miR-429 expression is upregulated in colorectal tissue and as such is closely related to tumor size, lymph node involvement and distant metastases, whereas it leads to shorter survival [102]. 

On the other hand, miR-145 does not only regulate angiogenesis but also other aspects of tumor progression and metastasis. For example, miR-145 was able to inhibit the expression of the protein c-MYC, which is involved in cell proliferation and is highly expressed in many types of cancer [103]. Additionally, it was found that miR-145 was able to downregulate the expression of MMP-9, which is involved in the degradation of extracellular matrix components and tumor invasion [104]. These results suggest that miR-145 may play an important role in regulating tumor angiogenesis and metastatic disease.

**Figure 2:**
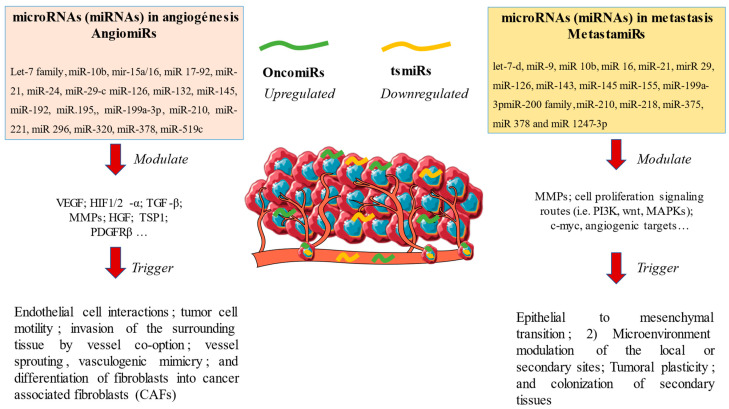
A general overview of the role of microRNAs in angiogenesis and metastasis. In this picture, different alterations in the up- or downregulation of oncogenic (OncomiRs) and onco-suppressive (tsmiRs) microRNAs cause alterations that lead to a stimulation of different mechanisms of angiogenesis and metastasis.

Other works have found that miR-218, miR-155 and miR-10 stimulate epidermal growth factors, TGFβ, MMPs and endothelial growth factors in addition to inhibiting the suppressor genes of metastases, such as EP300, acting together in such a way that the vascular invasion of tumor cells is promoted [105,106]. Other molecules such as miR-143, let-7-d and miR-126 stimulate the expression of oncogenes such as KRAS and therefore favor tumor growth and stimulate cell migration and metastatic invasion [107,108]. In addition, miR 155 favors an increase in cell permeability mechanisms through RhoA GTP, just as miR 9 is related to applications of the Myc gene and suppresses the expression of E cadherin, which promotes various mechanisms of cell invasion [109]. On the other hand, numerous miRNAs have been implicated in the EMT which implies a series of changes in the tumor matrix that favor tumor invasion and dissemination. For instance, mirR-29, miR- 21 and miR 10b can lead to the activation of ZEB, Twist or TGFβ, which are negative regulators of the transcription of the E cadherin gene [110]. Secondly, different miRNA alterations in the tumor microenvironment have been described in relation to bone metastases. An enhanced osteoclast differentiation is vital for metastatic proliferation at the bone level. In this sense, authors such as Ell et al. [111] demonstrated in cell lines of breast and bladder cancer how an overexpression of miR-16 and miR-378 promoted the activation of nuclear factor kappa B (NFkB), a marker of osteoclastogenesis related to a higher incidence of metastatic bone proliferation. In other lines of research, authors such as Fabbri et al. defined in lung cancer cell lines the roles of miR-21 and miR-29a in the activation of toll-like receptor-7 (TLR7) and TLR8, causing the release of interleukin 6 (IL-6) or tumor necrosis factor alpha (TNFα), which are proinflammatory mediators and favor tumor proliferation [112]. Similarly, authors such as Fang et al. have shown how an overexpression of miR 1247-3p leads to the activation of proinflammatory cytokines such as the proper IL-6 or IL-8 in hepatocarcinoma cell lines, favoring the transformation of fibroblasts and promoting changes that facilitate the formation of metastatic niches [113]. The relationship between miRNA and metastatic disease is complex and not fully understood. However, research has shown that miRNAs play an important role in the development and progression of metastatic cancer. Studies have found that miRNAs may be involved in the regulation of gene expression and cell signaling pathways, which can affect the spread of cancer cells to other parts of the body. The main miRNAs summarized in this section are collated in Table 3. Additionally, certain miRNAs may also be used as biomarkers for predicting the outcome of metastatic disease. In Figure 2, the mechanistic ways in which miRNAs influence the metastasis and angiogenesis/lymphangiogenesis processes are summarized. 

## 5. Conclusions

**Figure 3:**
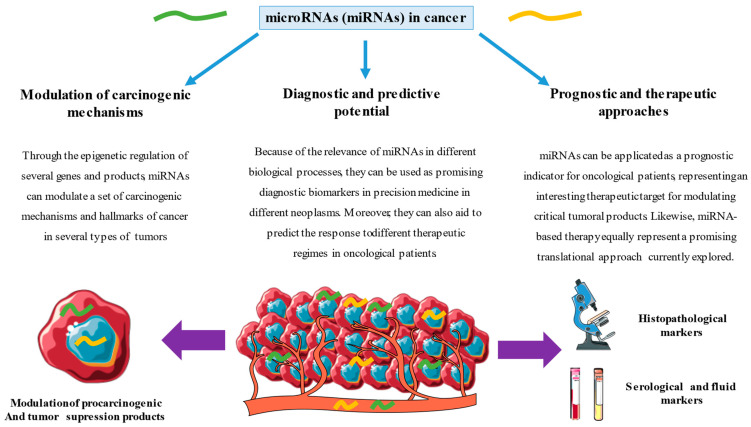
Roles and applications of microRNAs in cancer. Summary of possible applications of mi-croRNAs in explaining the pathophysiology of carcinogenesis and demonstrating usefulness in the diagnosis and prognostic assessment of different neoplastic diseases.

A growing body of evidence shows that miRNAs play a central role in the promotion of cell cycle alterations, angiogenesis and metastatic dissemination in a broad spectrum of neoplasms. Their study allows us to understand the underlying pathophysiology and expand our knowledge of these pathological metabolic pathways, which allows us to understand the complexity of the key points in tumor invasion. Concomitantly, and as represented in Figure 3, miRNAs represent a large set of molecules with promising applications as diagnostic, prognostic and predictive biomarkers, also representing attractive therapeutic targets and the prognosis of different neoplasms. However, further efforts are still warranted for linking and understanding the basic and biological roles of miRNAs and their clinical significance.

## Figures and Tables

**Table 1:** Main microRNAs implicated in cell cycle modulation, the type of cancer in which they are described and their expression and targets.

miRNAs	Type of Cancer	Expression	Targets Involved in Cell Cycle	Ref.
miR-497, miR-16, miR-30c-2-3p, miR-483-3p, miR-424, miR-543, miR-26a, miR-206, miR-15a, miR-30b, miR-708	Breast cancer	Downregulated	Cyclin E1, cyclin E2, cyclin D1, CDK1, CDK4, c-myc, E2F7, ERK/MAPK	[40]
miR-1207-5p, miR-492, miR-135b	Breast cancer	Upregulated	Cyclin D1, CDK2, c-myc, CDKN1A, CDKN-1B	[40]
17 oncomiRs and 39 tsmiRs	Lung cancer	Upregulated (oncomiRs) Downregulated (tsmiRs)	Cyclin D1, D2, E1, c-Myc, CDK2, CDK4, CDK6, Rb, p21, p53	[41]
Paralogous clusters of miR-17~92, miR-106a~363 and miR-106b~25	Colorectal cancer	Upregulated and downregulated	E2F and c-myc	[42]
tsmiRs: miR-124, miR-203 oncomiR: miR-221	Pancreatic cancer	Downregulated Upregulated	Rac1, survivin, p27	[23]
miR 221 and miR 222	Nasopharyngeal carcinoma	Upregulated	p27 kip1	[44]
miR-16	Prostate cancer	Downregulated	AKT3	[45]
tsmiRs: miR-145 OncomiRs: miR-223-3p, miR-107 and miR-21-5p	Penile cancer	Downregulated Upregulated	c-myc PTEN/MAPK signaling	[46]

**Table 2:** Main microRNAs implicated in angiogenesis and their expression and targets.

AngiomiRs	Expression	Targets Involved in Angiogenesis	Refs.
miR-21	Upregulated	VEGF, TGF-β, HIF-1α, MMPs, Ang-1	[52,53,54]
miR-126	Downregulated	VEGF, TSP-1	[55,56]
miR-199a-3p	Upregulated	VEGF, VEGFR, HGF, MMPs	[57]
miR-296	Upregulated	VEGFR2, PDGFRβ	[58,59,64]
miR-29-c	Upregulated	VEGF	[60]
miR-519c	Downregulated	HIF-1α	[62]
miR-145	Downregulated	HIF-2α	[63,65,66,67]
miR-30d and miR-323	Upregulated	VEGF	[64]
miR-182	Upregulated	HIF-1α	[64]
miR-146	Downregulated	EGFR and MMP2	[64]
miR-9 miR-181-5p	Downregulated Upregulated	MMP-14	[69,70]

**Table 3:** MetastamiRs and their expression and role in metastasis.

MetastamiRs	Expression	Implications in Metastasis	Refs.
miR-21	Upregulated	miR-21 enhances the ability of cancer cells to resist apoptosis and promote angiogenesis and tumor growth in metastatic disease. miR-21 expression was identified as being an independent prognostic biomarker for worse overall survival.	[76,77,110]
miR-210	Upregulated	MiR-210 is related with metastasis and poor survival in patients with breast cancer.	[78,79,80]
miR-199a-3p	Upregulated	Overexpressed in multiple types of metastatic cancers including hepatocellular carcinoma, colorectal cancer, non-small cell lung cancer and breast cancer. miR-199a-3p is involved in the modulation of the PI3K/Akt, MAPK and Wnt signaling pathways.	[81,82,83,84]
miR-9	Upregulated	Induces an aggressive tumor behavior by targeting several angiogenic and metastatic markers (i.e., PI3K and MAPKs).	[85,86,87,109]
miR-375	Downregulated	Targeting of several genes involved in the cascade of EMT.	[88,89,90]
miR-10b	Upregulated	Promotes cell migration and invasion through various target genes and pathways (PTEN/Akt, NF-κB signaling, RAS, DYRK1A and those involved in EMT).	[91,92,93,94,95]
miR-200 family (miR-200a, miR-200b, miR-200c, miR-141 and miR-429)	Downregulated in 10 types of cancer and upregulated in 2 cancer types	Exert a dual role in the invasion–metastasis cascade by suppressing EMT, tumor invasion and dissemination at the primary sites, but promote colonization in distant anatomic sites.	[96,97,98,99,100,101,102]
miR-145	Downregulated	Prevents metastatic disease by downregulating the expression of the protein c-MYC and MMP-9.	[103,104]
miR-218, miR-155	Upregulated	Stimulate epidermal growth factors, TGFβ, MMPs and endothelial growth factors in addition to inhibiting suppressor genes of metastases such as EP300, acting together in such a way that the vascular invasion of tumor cells is promoted.	[105,106]
miR-143, let-7-d and miR-126	Upregulated	These miRNAs stimulate the expression of oncogenes such as KRAS and favor tumor growth, cell migration and metastatic invasion.	[107,108]
miR-29	Upregulated	Activation of ZEB, Twist or TGFβ, limiting the transcription of the E cadherin gene.	[110]
miR 16 and miR 378	Upregulated	Activate NFkB which is a marker of osteoclastogenesis and is related to a higher incidence of metastatic bone proliferation.	[111]
miR 1247-3p	Upregulated	Favors metastasis via its effects on EMT and angiogenesis.	[113]

## Data Availability

Not applicable.
